# Supplementary material for: Spatially Resolved Optical Efficiency Measurements of Luminescent Solar Concentrators
Source: ACS Photonics. 2023 Jul 17;10(8):2886–93. doi: 10.1021/acsphotonics.3c00601 (PMC10436350; doi:10.1021/acsphotonics.3c00601)
Supplement: Supplementary file 1 — ph3c00601_si_001.pdf [file ph3c00601_si_001.pdf]

# Supplemental Information

Tomi K. Baikie, James Xiao, Bluebell H. Drummond, Neil C. Greenham, and Akshay Rao

*Cavendish Laboratory, J.J. Thomson Avenue, University of Cambridge, Cambridge, CB3 OHE, UK*

E-mail:

## Contents

**1 Supplementary Information** **2**

1.1 $\eta_{\text{int}}$ Method . . . . . . . . . . . . . . . . . . . . . . . . . . . . . . . 2

1.2 Integrating Sphere Design Rules . . . . . . . . . . . . . . . . . . . . . . . . 5

1.3 Laser Profile . . . . . . . . . . . . . . . . . . . . . . . . . . . . . . . . . 6

1.4 Calibration . . . . . . . . . . . . . . . . . . . . . . . . . . . . . . . . . . 7

1.5 $\eta_{\text{int}}$ Contribution from Secondary Photons . . . . . . . . . . . . . . . . . . . 9

1.5.1 $\eta_{collision}$ - Will an emitted photon collide with the LSC? . . . . . . . 11

1.5.2 $\eta_{\text{trans}}$ & $\eta_{\text{TIR}}$ - Is the photon transmitted into the LSC? . . . . . . . . 15

1.5.3 $\eta_{\text{abs}}$ - Can a photon be absorbed? . . . . . . . . . . . . . . . . . . . 18

1.5.4 $\eta_{\text{PLQE}}$, $\eta_{\text{escape}}$ . . . . . . . . . . . . . . . . . . . . . . . . . . . . 20
1.5.5 Summary . . . . . . . . . . . . . . . . . . . . . . . . . . . . . . . 21
1.6 $\eta_{\text{int}}$ Uncertainty Analysis . . . . . . . . . . . . . . . . . . . . . . . . . . . 21
1.7 Effective Path Length Correction . . . . . . . . . . . . . . . . . . . . . . . . 27
1.8 Subtended Angle Correction . . . . . . . . . . . . . . . . . . . . . . . . . . . 28
1.9 Pathlength, Angle Correction and Geometric Gain . . . . . . . . . . . . . . . 30
1.10 Single Value Decomposition . . . . . . . . . . . . . . . . . . . . . . . . . . 31
1.11 Determining $\eta_{EQE}$ . . . . . . . . . . . . . . . . . . . . . . . . . . . . . 32
1.12 $V_{\text{OC}}$ as a function of concentration . . . . . . . . . . . . . . . . . . . . . . 34
**References** **36**

# 1 Supplementary Information

## 1.1 $\eta_{\text{int}}$ Method

As detailed in the main text,

- for the first measurement, measurement A, the sphere is empty and laser light alone is measured. The spectral integral of the laser is proportional to the unabsorbed photons; we refer to this as $I_A$.
- For the second measurement, measurement B, the LSC is placed inside the sphere and

moved out of the beam path so the laser impinges on the sphere wall. Similarly, the spectral integral of the laser is $I_B$ and the photoluminescence profile is termed $E_B$.

- Measurement C, the laser is now directed onto the sample and care is taken to ensure the sample is oriented such that reflected laser light from the surface of the sample is directed into the sphere. The laser and PL spectral integrals are referred to as $I_C$ and $E_C$, respectively.

- In measurement D opaque material is applied to the edges of the LSC while the laser and sample orientation are the same as in measurement C. The opaque material will prevent emission from the edges of the LSC. As above, the laser and PL spectral integrals are $I_D$ and $E_D$, respectively.

In measurement B only $\mu$, the fraction of incident laser light scattered by the sphere wall *and* absorbed by the sample, will contribute to $I_b$ i.e.

$$I_b = I_a(1 - \mu). \tag{1}$$

For measurement C, an additional fraction, $(1 - A)$, of the incident light will be transmitted or reflected,

$$I_c = I_a(1 - A)(1 - \mu), \tag{2}$$

where $A$ represents the fractional absorption. From these two expressions, we may obtain an expression for the fractional absorption,

$$A = \left(1 - \frac{I_c}{I_b}\right). \tag{3}$$

The total number of photons, $I_c + E_c$ measured in measurement C is made up of two contributions; scattered laser light and chromophore emission due to the absorption of the laser light with efficiency $\eta_c$,

$$E_c = \underbrace{\eta_c' \ (1 - A) \ \mu \ I_a \ E_b}_{\substack{\text{contribution of} \\ \text{scattered laser light}}} \quad + \quad \underbrace{\eta_c \ I_a \ A.}_{\substack{\text{chromophore emission} \\ \text{due to absorption of laser}}} \tag{4}$$

As detailed extensively in the main text, $\eta$ is a function of illumination position. In **SI Equation 4** $\eta_c$ denotes the efficiency at the position of direct laser illumination, whereas $\eta_c'$ represents the efficiency arising from scattered laser illumination. The scattered laser illumination will impinge equally across the LSC and may represent a different average effective pathlength. However, where the fractional absorbance is high, as is targetted in most LSC designs, or where the $\eta$ is not position dependent, this disparity will be negligible. The fourth measurement, measurement D, will be the contribution of the entire LSC, $\eta_c$, minus the edges, $\eta_{\text{int}}$, contribution, i.e. $\eta_d = \eta_c - \eta_{\text{int}}$. Similarly to **SI Equation 4**, the total photon count in measurement D will be a combination of scattered and emitted light;

$$E_d = \eta_d' \ (1 - A) \ \mu \ I_a \ E_b + \eta_d \ I_a \ A. \tag{5}$$

The corresponding laser spectral integrals for measurements C and D are $I_c$ and $I_d$, respectively. Comparing **SI Equations 4** and **5** and including the laser integrals, we can write an expression for the optical efficiency of the LSC,

$$\eta_{\text{int}} = \frac{E_c - E_d + I_c - I_d}{I_a A}. \tag{6}$$

As long as laser fluctuations are small and calibration corrects for laser absorbance by the

opaque material, we can write $I_c = I_d$, and the expression for internal efficiency simplifies to

$$\eta_{\text{int}} = \frac{E_c - E_d}{I_a A}. \tag{7}$$

## 1.2 Integrating Sphere Design Rules

The size of the optic fibre, $d_o$, the acceptance angle of the fibre which is dependent on the numerical aperture, $N_a$, diameter of the integrating sphere, $d_I$, the position of the baffle along the radius of the sphere, $d_b$ and the size of the LSC, $d_{LSC}$, determine the minimum size of the baffle. The aim is to ensure the no direct illumination from the sample. The acceptance angle of the fibre is given by

$$\theta = \sin^{-1}\left(\frac{N_a}{N_0}\right), \tag{8}$$

where $N_0$ is the refractive index of air. The minimum length of baffle can be determined by solving the distance between the bounds of the arc centered at the optical fibre port at radius less than $d_I$.

A secondary constraint is determined by the base of the isosceles triangle formed between the optical fibre port and the surface of the LSC. Although the optical fibre will not accept photons at this angle, by design, we prevent direct illumination of the optical fibre by the LSC. Therefore, the baffle must always be larger than the shortest line segment of the triangle to prevent direct illumination. As two bounces must occur to satisfy the Lambertian reflectance, the baffle must always be larger than both constraints. Two examples are given for two geometries in **SI Figure 1**, noting the impact of $N_a$ on sphere design. The baffle utilised in the reported measurements has a radius of 10 cm and is placed 8 cm from the

optic fibre port, which is greater than the required baffle size, including when accounting for LSC movement within the sphere.

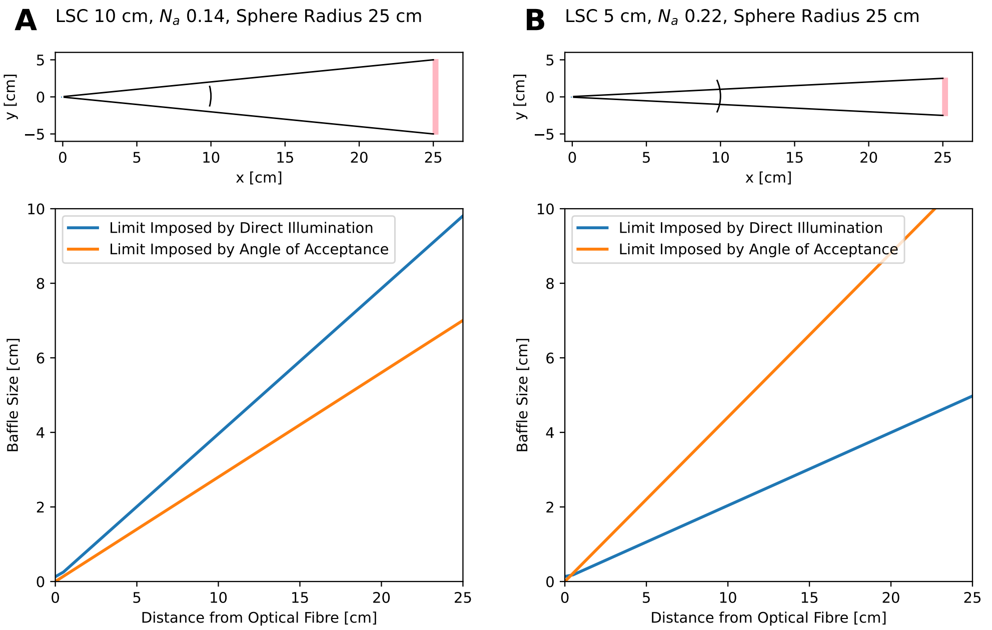


Figure 1: Cartoon representing the internal geometry of the integrating sphere. Pink cuboid represents LSC dimensions. The black arc represents the angle of acceptance of the optic fiber. Black lines are direct illumination paths from the LSC to the optic fiber port. The constraints on baffle size are plotted for two scenarios. The baffle must be larger than both the blue and orange line. **A** - Outlines the case where $d_I = 50$ cm, $d_{LSC} = 10$ cm and $N_a = 0.14$, and **B** outlines the limiting case for $d_I = 50$ cm, $d_{LSC} = 10$ cm and $N_a = 0.22$.

## 1.3 Laser Profile

The beam profile of the laser was approximately rectangular, 3000 $\mu$m by 1000 $\mu$m, power was recorded to be 10 mW, giving an excitation density of 3.3 mW per mm$^2$. 30 minutes was given to stabilise the laser before measurement, as observed in **SI Figure 2** to reduce fluctuations between different measurements, which would impact measured efficiency values.

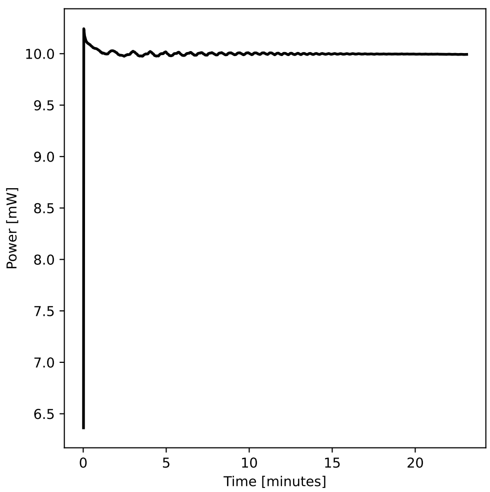


Figure 2: Power of laser as a function of time, highlighting the variation in power until laser has stabilised.

## 1.4 Calibration

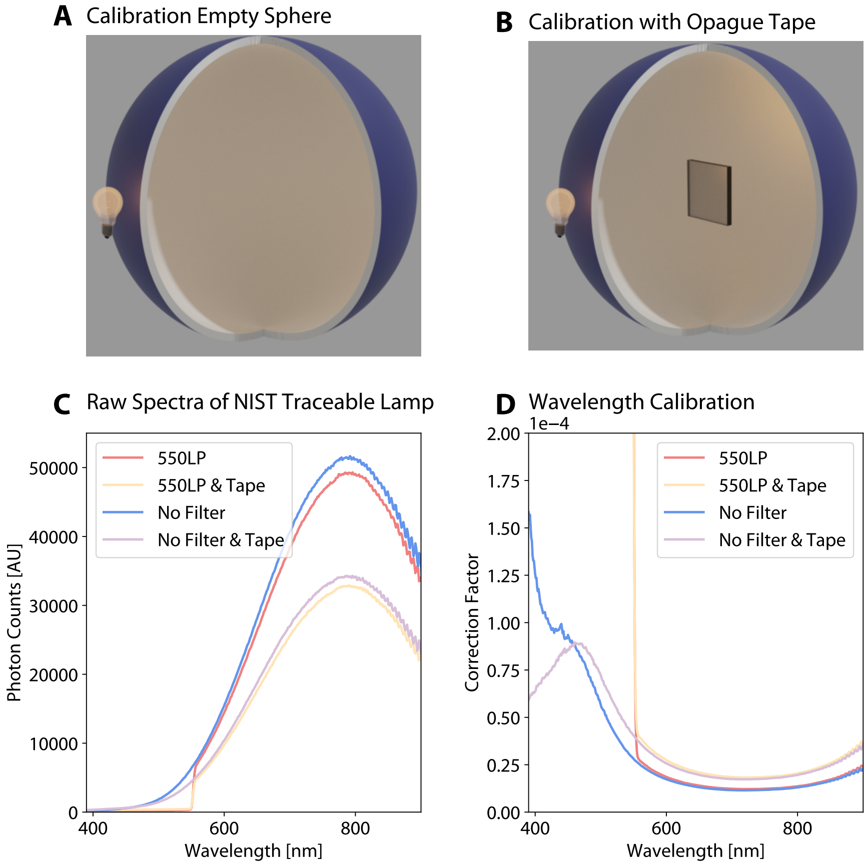


Figure 3: **A** - The calibration lamp is allowed to illuminate the sphere, and **B** - the calibration lamp illuminates a transparent sample, with the edges covered, allowing for quantification of the absorbance of the opaque layer. **C** - outlines the measured spectra, which are then compared to the known spectra of the lamp resulting in **D** - calibration files.

The spectrometer used for optical efficiency measurements is required to be calibrated for spectral response with a source with a known spectrum. Allowing for the limitations of detector sensitivity and saturation, this calibration must be performed with the same acquisition settings as the measurement as to not alter the detector response of the spectrometer. In this case, acquisition settings primarily signify monochromator parameters such as slit sizes, diffraction grating blaze,and other apertures controlling signal intensity. Thus a series of calibration spectrums are created for each slit size, long pass filter and aperture size.

For the calibration measurement, the calibrated light source is placed in the integrating sphere port and aligned toward the detector. Care must be taken in order to align the source directly into the sphere in order to minimise any errors from possible angular dependence of lamp emission. A 200 W NIST-traceable QTH lamp (Newport 63976 200QC OA) was used for the purpose of calibration of the integration sphere. In the case of the optical efficiency measurements, calibration requires at least two measurements to account for the absorption of the opaque material applied to the edges of the LSC. The first of the calibration files are then applied to the recovered spectrum from measurements A, B, C and then the second to measurement D. Another calibration with 3 edges covered is used for the spatially resolved measurements where one edge is uncovered.

The calibration source will also contain a specified degree of wavelength dependent uncertainty which is converted into a systematic error once embedded within a sensitivity curve. For a quality lamp, the expanded uncertainty may be as high as 2% for the extremes of the spectrum and as low as 0.5% for the middle.[1]

## 1.5 $\eta_{int}$ Contribution from Secondary Photons

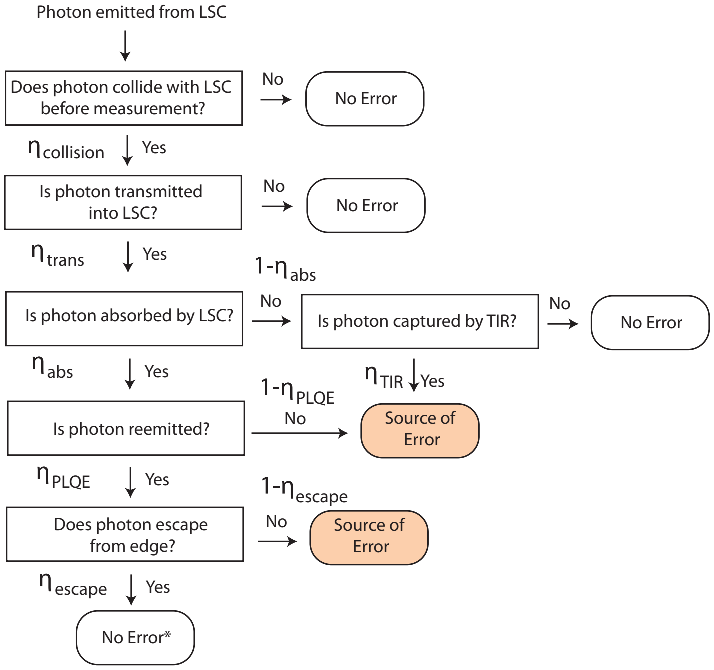


Figure 4: Origins of $\eta_{int}$ from secondary photons in photoluminescence measurements in integrating spheres. Photons which are transmitted to the edges altering the edge photoluminescence or photons which are reabsorbed before detection will contribute to $\eta_{int}$.

The probability of an emitted photon inducing an error in $\eta_{int}$ is outlined in **SI Figure 4**. Firstly, we determine what portion of photons, $\eta_{collision}$, that leave the LSC collide with the LSC before measurement. If photons are reflected from the LSC, there is no contribution to the error.

However, if the photons are transmitted into the LSC, with efficiency $\eta_{trans}$, there is an opportunity for these photons to be absorbed with probability $\eta_{abs}$. The probability of absorption will be a function of the overlap of photoluminescence, absorption and the distance the photon travels in the LSC.

Even if the photon is not absorbed, it may be captured by total internal reflection ($\eta_{TIR}$) and be edge emitted, which would result in an increase of measured edge emission and

an associated error. If the photon is absorbed, it may decay through some non-radiative pathway, which would lower measured counts, also inducing error. If the photon is emitted and then travels to the edge, this would also contribute to the error. However, if the photon leaves through the top surface, as this is corrected for in the all edges covered measurement, ir result in no magnitude changes of the integrated counts, which is of relevance in determining $\eta_{\mathrm{int}}$, however, it may alter the spectral shape by red-shifting the signal.

We address each of these probabilities in turn, attempting to determine the error associated with secondary photons. $\eta_{collision}$ is a function of sphere and LSC size, in an integrating sphere $\eta_{\mathrm{trans}}$ and $\eta_{\mathrm{TIR}}$ are functions of the refractive index of the LSC. $\eta_{\mathrm{abs}}$ is a function of the size of the LSC and chromophore concentration.

### 1.5.1 $\eta_{collision}$ - Will an emitted photon collide with the LSC?

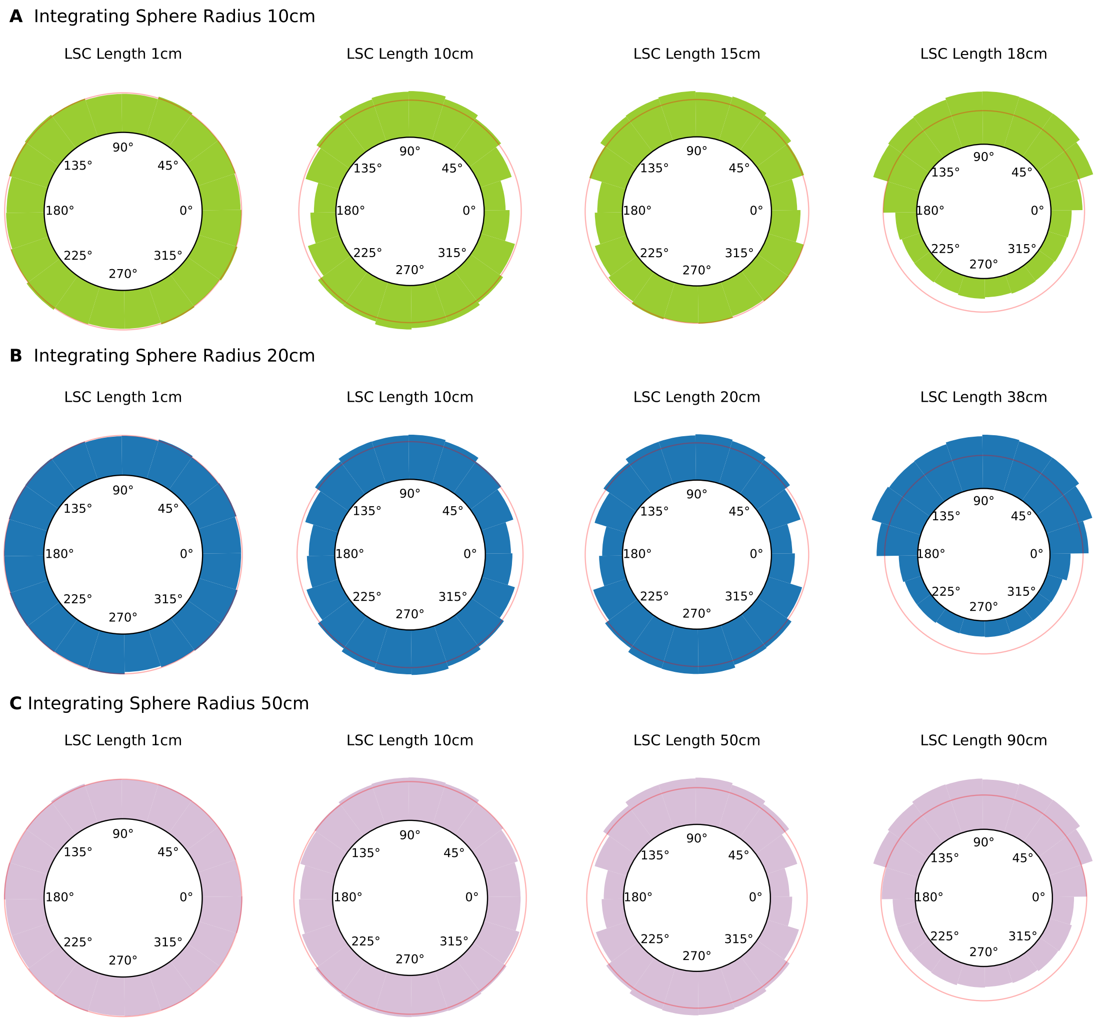


Figure 5: A histogram of photon locations after 100 bounces, highlighting how the photon distribution changes because of the relationship between LSC size and sphere size. The orange line indicates an equal distribution across the entire sphere.

To understand the error associated with secondary reabsorption, we utilise a Monte Carlo ray tracing algorithm we can determine how many photons will interact with the LSC as a function of sphere radius and LSC size. Supposing a point just above the LSC along the direction of laser illumination emits randomly, the test is to determine what number of photons are reabsorbed and thus contribute to $\eta_{\text{int}}$ error. The two dimensional simplification will give a upper bound for allowed geometries compared to the three dimensional case.

For reasons of computational efficiency, we develop a vectorised approach. Let the sphere be defined its centre $c$ and radius $r$. $p_0$ is the point of isotropic emission with the direction of emission given by $v = (\cos[\text{RandomReal}], \sin[\text{RandomReal}])$, where RadomReal is a random value between 0 and 1. The displacement vector from the centre to the point is given by $u = p_0 - c$. Therefore the intersection of the photon with the sphere, $p_1$ is defined by

$$p_1 = p_0 + \frac{(n-d)}{\|v\|} v, \tag{9}$$

where the scalars $n$ and $d$ are given by

$$d = \frac{u.v}{\|v\|}, n = \sqrt{r^2 + d^2 - u.u}. \tag{10}$$

The intersections, if they exist, between the photon ray defined by $p_0$ to $p_1$ and the 4 lines formed by the perimeter of the LSC are then determined. If these intersections with the LSC exist, the dot product between the intersection and $p_0$ is determined to check the direction of the ray. The intersection which has the smallest euclidean distance to $p_0$ is chosen. If the intersection is with the LSC perimeter the photon "dies" and the next photon is run. If the intersection with the sphere is recorded, a new direction vector is determined by randomly selecting a vector which keeps the ray within the integrating sphere, i.e. $-\frac{\pi}{2} < \theta < \frac{\pi}{2}$ and the code is repeated with $p_0 = p_1$.

To determine the number of bounces to be modeled, we approach the problem analytically and solve for the number of bounces before being detected, known as the sphere multiplier, $M$,

$$M = \frac{\rho}{1 - \rho(1-f)} \tag{11}$$

where $\rho$ represents the reflectance of the coating, and $f$ represent the output port area fraction relative to the internal sphere area, i.e. $f = (A_{\text{entrance}} + A_{\text{exit}})/A_{\text{sphere}}$. For our experiment the port fraction is $f \leq 0.01$, with sphere radius of 25 cm and two ports no larger than $r = 5$ cm. The interior sphere coating is barium sulphate, which is reported to have a reflectivity of $\rho = 0.992 \pm 0.001$.[2]

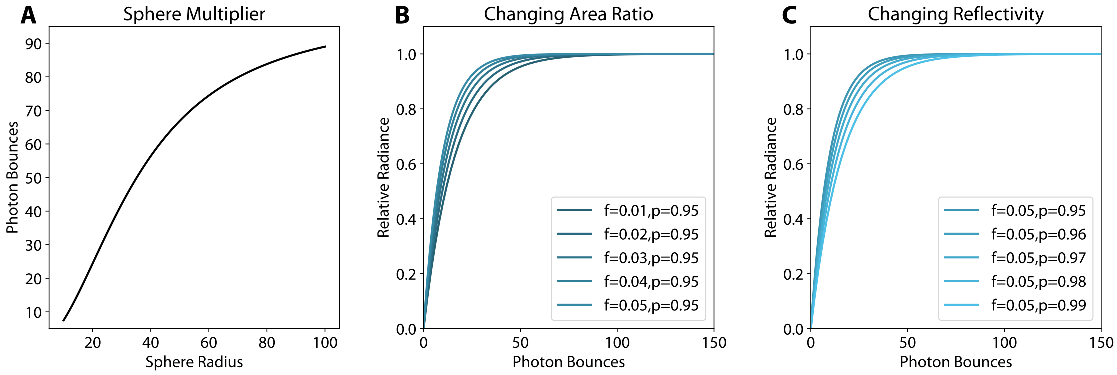


Figure 6: **A** - Sphere multiplier, the average number of photon bounces before being detected, as a function of sphere radius. **B & C** - Plots of the relative radiance for a sphere of radius 25 cm for changing reflectivities and input and exit port sizes. Relative radiance is the ratio of the radiance after $n$ bounces divided by the radiance of the steady state solution, as $n \to \infty$. The sphere multiplier defines the number of photon bounces before measurement and the relative radiance defines the number of bounces required for a uniform distribution in the spectrum across the internal surface of the sphere.

To determine if this sphere multiplier represents a steady state of the sphere, let $\phi$ represent the input flux, After $n$ reflections, the total flux, $F$, incident over the internal integrating sphere surface is given by the power series,

$$F = \phi \sum_{n=1}^{n} \rho^n (1 - f)^n, \tag{12}$$

as $\rho(1 - f) < 1$. In the infinite reflection limit, we can write

$$F = \frac{\phi \rho (1 - f)}{1 - \rho(1 - f)}. \tag{13}$$

We define the relative radiance as the ratio of the radiance after $n$ bounces divided by the radiance of the steady state solution, as $n \to \infty$. **SI Figure 6** gives the relative radiance for a series of different $f$ and $\rho$. We find that $n \approx 30$ bounces is sufficient to describe the steady state within the sphere in our case, which is similar to the sphere multiplier.

Returning to the ray tracing model, we now model the number of photons which interact with the LSC before the number of bounces given by the sphere multiplier, which is the average number of bounces before detection. This is given as a function of LSC size and radius in **Figure 2C**. This is 94% for a sphere of radius 25 cm and LSC width of 10 cm in the 2D approximation we make here. In the 3D case, this value will be smaller, as the square LSC takes up a smaller proportional area.

### 1.5.2 $\eta_{\text{trans}}$ & $\eta_{\text{TIR}}$ - Is the photon transmitted into the LSC?

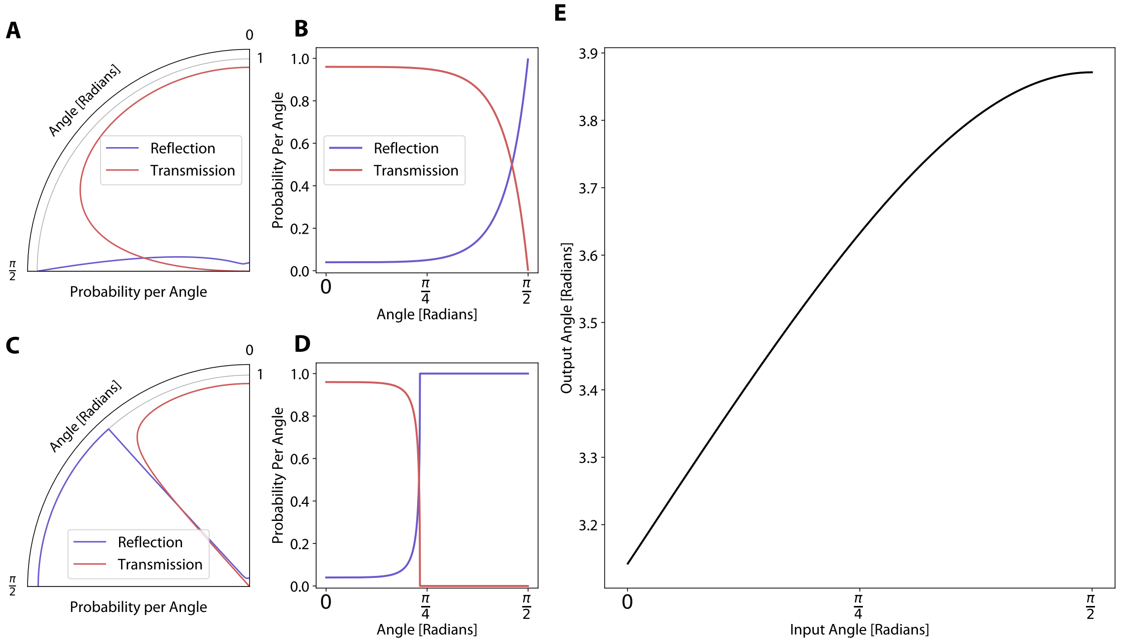


Figure 7: **A** - Probability per angle of a photon being reflected or transmitted when incident on the LSC plotted on polar axis, **B** - equivalent to A, however the angle is plotted on a linear axis. **C** - Probability per angle of a photon being reflected or transmitted when leaving the LSC, plotted on polar axis, **D** - equivalent to C, but angle is plotted on a linear axis. **E** - Change of angle with respect to the surface normal when photon is refracted at the first interface.

We analytically determined the probability of reflection and transmission for each angle, from the well known Fresnel equations.[3] We then determined the probability of reflection and transmission for both the incident beam, moving from $n_1$ to $n_2$, where $n_1 < n_2$ (**SI Figure 7A&B**), and the beam of photons within in the LSC, moving from $n_2$ to $n_1$ (**SI Figure 7C&D**). We also related every photon incident to the LSC at every angle directly to the new refracted beam within the LSC, with respect to the top surface normal, see **SI Figure 7E**.

In the integrating sphere, secondary photons are isotropically scattered throughout the integrating sphere, and so we assume a constant probability distribution function (PDF), which approximates the isotropic secondary photon distribution within the integrating sphere. This

is represented by the green area in **SI Figure 8A**, and integrates to 1 over $\theta = 0$ to $\pi/2$. Multiplying this by the probability of transmission into the LSC (red line, **SI Figure 7A**), gives the PDF for transmission represented by the blue area in **SI Figure 8A**. Integrating this over all angles returns 0.86, meaning 86% of photons are transmitted into the LSC, assuming $n_1 = 1$ and $n_2 = 1.5$.

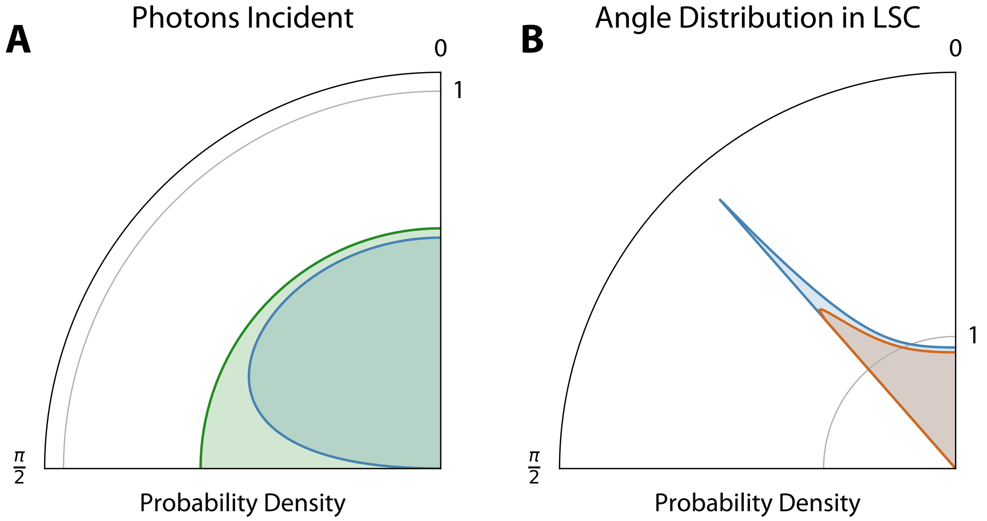


Figure 8: **A** - Probability distribution function of photons from 0 to $\pi/2$. Green area represents photons impinging on the LSC isotropically. The blue area is the portion which will be transmitted. Angles are with respect to the top surface normal. **B** - After transmission, photons will be refracted. The blue region area matches the area of the blue area in A. A portion, outlined in red, of these photons will now be transmitted again. Angles are now with respect to the bottom surface normal, pointing into the LSC.

If the photons are transmitted, they will also be refracted, and the angle with respect to the normal change according to **SI Figure 7E**. For any LSC with parallel planes, referencing the transmitted angles to the back surface normal of the LSC, is simply a correction of $-\pi$. However, the transmission process induces a coordinate transformation, as the spacing of the angles have changed. As such, we must to account for the change of variable in a manner analogous to a Jacobian transformation.[4] Representing the coordinate transformation by $g : \mathbb{R} \to \mathbb{R}$, and as it a monotonic function (by inspection, **SI Figure 7E**), then the resulting density function is given by

$$f_Y(y) = f_X\left(g^{-1}(y)\right)\left|\frac{d}{dy}\left(g^{-1}(y)\right)\right|, \tag{14}$$

where $g^{-1}$ denotes the inverse function. The blue region in **SI Figure 8B** represents the same photons as the blue area in **SI Figure 8A** after undergoing the coordinate transformation. Reassuringly, the integral over all angles remains 0.86. This PDF may then be multiplied by the probability per angle of transmission out of the LSC, as in **SI Figure 7C&D**. The red region, which corresponds to 77% of the photons, represents the directly transmitted photons, the remainder of 9% are reflected back into the LSC. Any photons which are reflected back into the LSC we assume contribute to $\eta_{\text{int}}$ error by either being absorbed or by travelling to the edge. Here we make the explicit assumption that all photons are which are reflected from the bottom surface remain in a TIR mode, which is a worst case assumption. **SI Table 1** summarises these results, which are general for LSCs of any dimension and dependent only on the refractive index of the LSC, assuming the constant PDF and parallel surfaces.

Table 1: Assuming constant PDF and parallel surfaces, probabilities of photons being transmitted and reflected.

| Photons that Impinge | Transmission Top | Transmission Bottom | Reflected Bottom |
|---|---|---|---|
| 1 | 0.86 | 0.77 | 0.09 |

### 1.5.3 $\eta_{\mathbf{abs}}$ - Can a photon be absorbed?

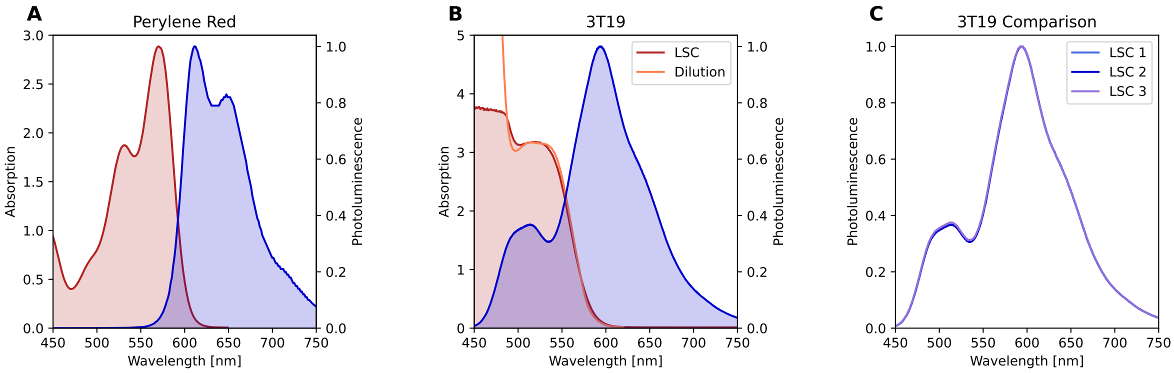


Figure 9: **A** - Red region is the linear absorption of the 0.4 mM perylene red LSC. The normalised photoluminescence is given in blue. Both are measured outwith the integrating sphere. **B** - Similarly for the 3T19 LSC. The absorption of the 3T19 LSC reached the instrumentation limit, therefore a dilution of the LSC was made by dissolving part of the LSC in chlorobenzene. **C** - Comparison of the 3519 LSCs sourced from different suppliers.

To approximate the probability of reabsorption, we measured the linear absorption and photoluminescence of the LSC outwith the integrating sphere (see **SI Figure 9**). This measurement includes any LSC matrix absorption. We are interested in the portion of the photoluminescence that may be absorbed. In the case off the perylene red LSC, we assume any photon with wavelength less than 620 nm may be absorbed which corresponds to the wavelength that is 99% of the peak absorbance. Integrating the photoluminescence probability distribution function, from $\lambda = 0$ to $\lambda = 620$ nm which corresponds to 0.31, meaning 31% of photons emitted may be reabsorbed. This actual number of photons absorbed will be dependent over the path length the absorption occurs.

The Beer-Lambert law relates absorption to the optical path length as

$$A = \epsilon l c = \log_{10}\left[\frac{I_0}{I}\right], \tag{15}$$

where $A$ is the absorbance, $\epsilon$ is the attenuation coefficient, $l$ is the length of the optical path

and $c$ is the concentration. Following **SI Equation 15**, the portion of photons absorbed is then $1 - 10^{-A}$.

We are interested in the pathlength of photons that can be reabsorbed, for example, in the perylene red LSC we suppose that any photon with wavelength less than 620 nm may be absorbed. By integrating the PDF of the perylene red photoluminescence, we may obtain an average absorbance over this region of 0.47, which corresponds to all photons that may be reabsorbed. This may be approximately verified by the average absorbance over the union of the photoluminescence and absorbance in **SI Figure 9A**.

In the linear measurement, the pathlength measured in the linear absorbance measurement is the thickness of the LSC, which is $l = 2$ mm and the concentration is 0.4 mM, we can determine $\epsilon = 0.59$ per mm per mM. However, in the integrating sphere, the path length will change as photons are not impinging uniformly parallel to the surface normal.

This pathlength can be easily determined the average angle that a transmitted photon is impinging on the bottom surface, which is simply the angular expectation value, E,

$$\mathrm{E}[x] = \int_0^{\pi/2} x f(x) dx = 0.3 \text{ rad}, \tag{16}$$

where $f(x)$ is defined by **SI Equation 14**. The average path length, $\bar{l}$, for the photon beam which would otherwise be transmitted and is impinging on the LSC isotropically then

$$\bar{l} = \frac{d}{\sin(\frac{\pi}{2} - 0.3)}, \tag{17}$$

where $d$ is the thickness of the LSC, 2 mm in this case. Returning to **SI Equation 15**, we may now determine the absorbance over this pathlength, and the absorbance is $A = 0.5$. This corresponds to 70% absorption of transmitted photons that may be absorbed. Therefore, in

the case of the perylene red LSC, this is $\eta_{\text{abs}} = 0.7 \times 0.31 = 0.22$. In the 3T19 LSC case, we obtain that 0.54 may be absorbed, and even though $c$ is unknown, as $\epsilon c$ is constant, we obtain $A = 1.98$ meaning $\eta_{\text{abs}} = 0.54 \times 0.99 = 0.54$

**SI Figure 9C** outlines normalised photoluminescence of the 3T19 from different suppliers. LSC 1 was purchased from Bay Plastics Limited (Tyne and Wear) and laser cut. LSC 2 was purchased from Plastic Online (Kingston upon Hull) and polished. LSC 3 from Perspex Sheet UK (Leicester) and saw cut. The front face was measured, and photoluminescence from each supplier was consistent.

#### 1.5.4 $\eta_{\text{PLQE}}$, $\eta_{\text{escape}}$

Methods for establishing $\eta_{\text{PLQE}}$ are well established.[5,6] Perylene Red has a reported photoluminescence quantum efficiency of 95%.[7] The escape cone is defined by rays that intersect the LSC surface at angles smaller than the escape angle, $\theta_{\text{escape}}$, given by

$$\theta_{\text{escape}} = \sin^{-1}\left(\frac{n_0}{n_1}\right). \tag{18}$$

We make the assumption that for an isotropically emitting chromophore, the per emission escape loss rate equals the proportion of the solid angle defined by some critical cone out of the full solid angle of the sphere, giving an efficiency of

$$\eta_{\text{escape}} = 1 - \sqrt{1 - \left(\frac{n_0}{n_1}\right)^2} = 0.25 \tag{19}$$

A comprehensive analysis of escape-cone losses for different geometries and accounting for photo selection and polarization is given elsewhere.[8,9] Further, only if the reabsorbed photon is lost due to a non-unity quantum yield or absorbed by the opaque edge, will the reabsorbed

photon alter the $\eta_{\text{int}}$, as red-shifting does not alter the spectral integral. Therefore, if $\eta_{\text{PLQE}}$ and $\eta_{\text{escape}}$ cannot be determined directly, as an approximation, the measured $\eta_{\text{int}}$ may be used.

### 1.5.5 Summary

As summarised in **SI Table 2**, for our experimental set-up, 96% of photons will intersect with the LSC. Assuming all secondary photons are impinging the LSC isotropically, with a PDF matching that depicted in **SI Figure 8A**, then 86% will be transmitted and 6% reflected if they are not absorbed. In the case of the perylene red LSC, the maximum contributed associated with secondary photon reabsorption is

$$\begin{aligned}\eta_{\text{r}} &\leq \eta_{\text{collision}}\eta_{\text{trans}}\Big((1-\eta_{\text{abs}})\eta_{\text{TIR}} - \eta_{\text{abs}}(1-\eta_{\text{PLQE}}) + \eta_{\text{abs}}\eta_{\text{PLQE}}(1-\eta_{\text{escape}})\Big) \\ &\leq 0.17\end{aligned} \tag{20}$$

Table 2: Summary of pathways leading to secondary photon contribution to $\eta_{\text{int}}$

| LSC | $\eta_{\text{collision}}$ | $\eta_{\text{trans}}$ | $\eta_{\text{TIR}}$ | $\eta_{\text{abs}}$ | $\eta_{\text{PLQE}}$ | $\eta_{\text{escape}}$ |
|---|---|---|---|---|---|---|
| Perylene Red | 0.94 | 0.86 | 0.09 | 0.22 | 0.95 | 0.25 |
| 3T19 | 0.94 | 0.86 | 0.09 | 0.54 | - | - |

## 1.6 $\eta_{\text{int}}$ Uncertainty Analysis

Assuming a perfect measurement with no laser fluctuation, or calibration source variation, the expression for the optical efficiency is given in the main text and reproduced here,

$$\eta_{int} = \frac{E_c - E_d}{I_a\Big(1 - \frac{I_c}{I_b}\Big)}. \tag{21}$$

Every measured term, $C \in \{I_a, I_b, I_c, E_c, E_d\}$, is the sum of calibration corrected detector counts, i.e.

$$C = \sum_{n=1}^{\text{pixels}} a_n x_n, \tag{22}$$

where $a_n$ is the spectral sensitivity (as detailed in **SI Section 1.4**) and $x_n$ is the detected counts at pixel $n$. The absolute error, $u_c$, of the integrated photon count is the error from each pixel,

$$u_c = \left( \sum_{n=1}^{\text{pixels}} a_n^2 u_n^2 \right)^{1/2}, \tag{23}$$

where $u_n$ is the absolute uncertainty, which is made up from of detector read noise, $u_r$, and shot noise, $u_p$ for each pixel. The read noise $u_r$ is dependent on acquisition settings. For each measurement we subtract a background measurement. As the subtraction involves two values with themselves having associated errors, the measured error is added in quadrature. As the acquisition settings are identical, the standard error may be regarded as the same in both acquisitions. Therefore the standard error, $u_r$ is multiplied by a factor of $\sqrt{2}$. This gives the error present following background subtraction. We determined the read noise by measuring the variation over sequential dark measurements, and found the number of counts to vary by 60 counts per pixel for a 40 second measurement.

Measured signals will also be affected by shot noise. The standard error of shot noise arising from random fluctuations in discrete events is equal to the square root of the number of events;

$$u_p = x_i^{1/2}. \tag{24}$$

For a CCD or photodiode detection methods with non-unity quantum efficiencies, the noise is equal to the square root of the number of detection events, rather than the number of incident photons, having been corrected for quantum efficiency. Thus the absolute value of shot noise error is decreased but the signal to noise ratio is also decreased. Thus, the uncertainty from shot noise and read noise, accounting for background correction, is equal to the quadrature sum of both;

$$u_i = \sqrt{2u_r^2 + u_p^2} = \sqrt{2u_r^2 + x_i}. \tag{25}$$

Thus, the total error in the photon count, $u_c$, becomes

$$u_c = \left( \sum_{n=1}^{\text{pixels}} a_n^2 (2u_r^2 + x_n)^2 \right)^{1/2}. \tag{26}$$

Any detected signal is proportional to the incident laser excitation fluence. The percentage error in laser output can be converted into an absolute error by multiplying the measured photon count by the percentage fluctuation error of the laser, $l$, defining

$$u_l = l\ C. \tag{27}$$

Now, our error becomes the quadrature sum of the absolute laser error and **SI Equation 26**,

$$u_{c,l} = \sqrt{u_c^2 + l^2 C^2}. \tag{28}$$

For the photoluminescence measurements, the error associated with secondary reabsorption by the LSC, $u_r$, is additionally a function of the reabsorption probability as defined in

**SI Equation 20**, and the total error becomes

$$u_{c,l,r} = \sqrt{u_c^2 + l^2C^2 + \eta_r^2C^2}. \tag{29}$$

Let

- $u_{I_a}$ be the error with respect to measurement $I_a$,
- $u_{I_b}$ be the error with respect to measurement $I_b$,
- $u_{I_c}$ be the error with respect to measurement $I_c$,
- $u_{I_d}$ be the error with respect to measurement $I_d$
- $u_{E_b}$ be the error with respect to measurement $E_b$,
- $u_{E_c}$ be the error with respect to measurement $E_c$,
- $u_{E_d}$ be the error with respect to measurement $E_d$,

and neglecting covariance in the error between individual measurements, the standard error in $\eta_{\text{int}}$, $u_\eta$, becomes the quadrature sum of the absolute errors multiplied by the partial derivative of the $\eta_{\text{int}}$ expression for each expression[10,11]

$$u_\eta = \left(\left(\frac{\partial\eta}{\partial E_c}\right)^2 u_{E_c}^2 + \left(\frac{\partial\eta}{\partial E_d}\right)^2 u_{E_d}^2 + \left(\frac{\partial\eta}{\partial I_a}\right)^2 u_{I_a}^2 + \left(\frac{\partial\eta}{\partial I_b}\right)^2 u_{I_b}^2 + \left(\frac{\partial\eta}{\partial I_c}\right)^2 u_{I_c}^2\right)^{1/2}. \tag{30}$$

Where

$$\frac{\partial\eta}{\partial E_c} = \frac{1}{I_a\left(1 - \frac{I_c}{I_b}\right)}, \tag{31}$$

$$\frac{\partial \eta}{\partial E_d} = -\frac{1}{I_a\left(1 - \frac{I_c}{I_b}\right)}, \tag{32}$$

$$\frac{\partial \eta}{\partial I_a} = -\frac{E_c - E_d}{I_a^2\left(1 - \frac{I_c}{I_b}\right)}, \tag{33}$$

$$\frac{\partial \eta}{\partial I_b} = -\frac{I_c\left(E_c - E_d\right)}{I_a I_b^2\left(1 - \frac{I_c}{I_b}\right)^2} \tag{34}$$

and

$$\frac{\partial \eta}{\partial I_c} = \frac{E_c - E_d}{I_a I_b\left(1 - \frac{I_c}{I_b}\right)^2}. \tag{35}$$

We find several situations where the measurement noise may contribute to large uncertainty in $\eta_{\text{int}}$ measurement may arise: low excitation powers, poor photoluminescence quantum efficiency, very high and especially very low absorption. Measurements with inadequate signal-noise ratio at low excitation powers and/or poorly emitting samples can be improved by increasing acquisition time through multiple cumulative exposures with longer exposure times, as background-corrected read noise becomes dominated by absolute shot noise or laser fluctuation errors. Extremely absorbing samples can produce low signal for $I_c$, which is compounded if the sample also has low $\eta_{\text{int}}$, resulting in low PL signals and read noise dominating. Samples with very low absorption have greater uncertainty, where the difference between $I_c$ and $I_b$ terms are small leading to the denominators in their respective derivative terms approaching zero, resulting in inflated factors for their error terms.

Broadly, luminescence terms are typically dominated by read noise, whilst the laser terms are more susceptible to shot noise, as laser terms typically consist of high number of counts integrated across a small number of pixels, whilst luminescence terms typically consist of

fewer counts integrated across a large number of pixels. Practically, shot noise is limited by the dynamic range of the detector. Notably, the stability of the excitation source affects all measurements and increases the minimum uncertainty for the resulting $\eta_{\text{int}}$. Laser fluctuation increases error for all measurements and therefore tends to dominate over read noise or shot noise.

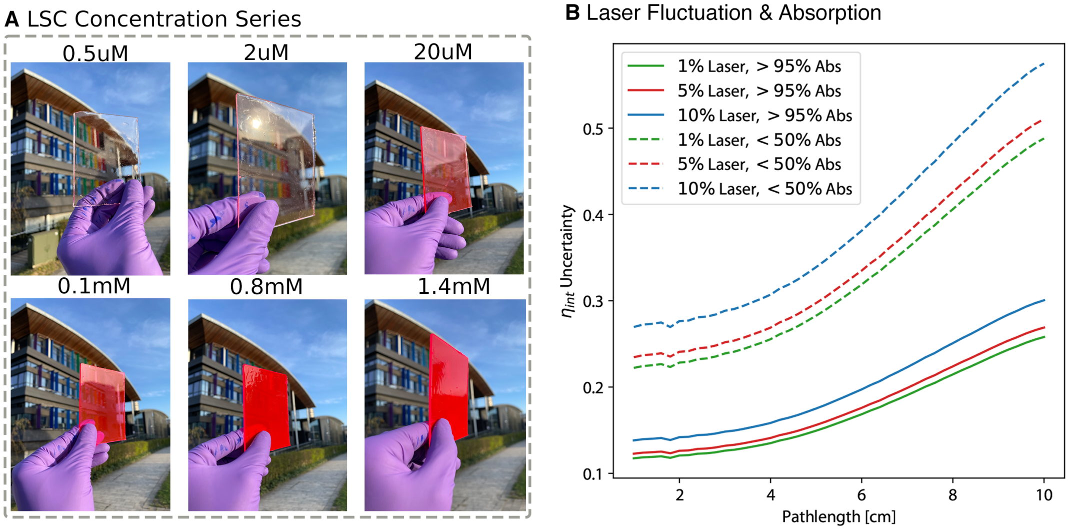


Figure 10: Concentration Series and Uncertainties. **A** - Photographs of the produced LSCs at different concentrations outside the Maxwell centre highlighting changing optical densities. **B** - Plot of error in $\eta_{\text{int}}$ as a function of pathlength with changing laser fluctuation (1%, 5% or 10%) and 95% absorption and 50% absorption.

## 1.7 Effective Path Length Correction

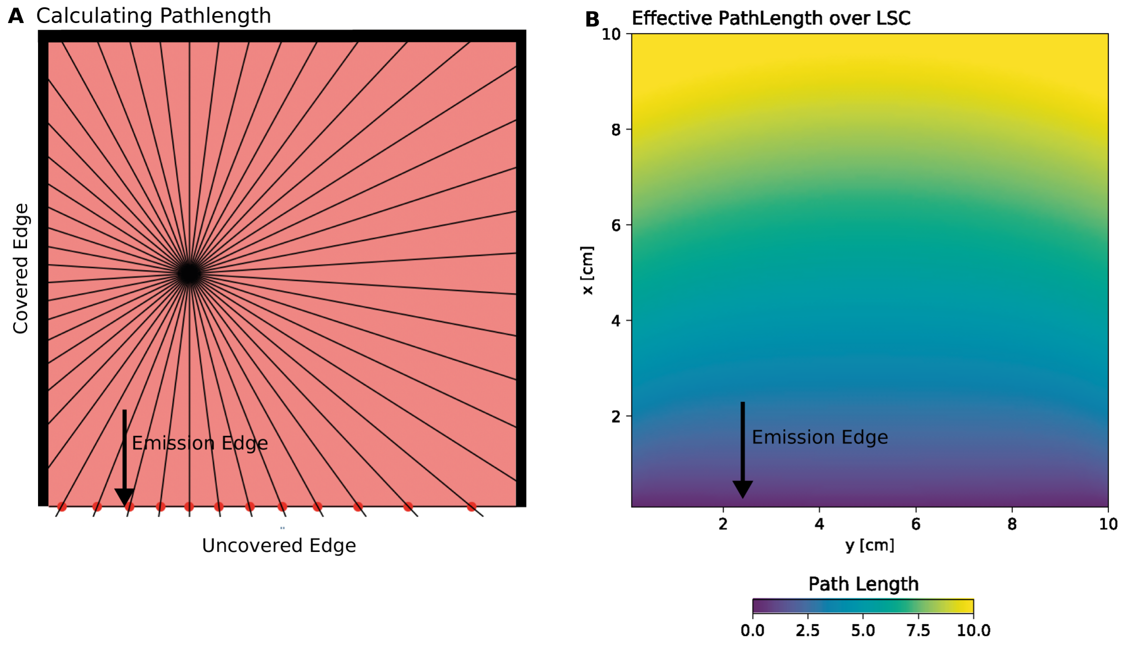


Figure 11: **A** - Cartoon describing the solid angle problem. The aim is to calculate the average length of the black line, the effective path length, from the illumination point to the uncovered illumination edge, highlighted with red dots. **B** - The effective pathlength, which is a plot of **SI Equation 37**. The distance along the dimensions of the LSC is given by $x$ and $y$, where $y = 0$ corressponds to the emission edge.

The effective path length, $\bar{l}$, is defined as the average distance from the illumination point, to the uncovered edge. It is distinct from the actual path length, which is often recovered from a ray-tracing simulation, which will account for the real distance a photon travels due to reflection events. Let $(p_x, p_y)$ define the illumination point and $L$ represent the length of one edge of the LSC. In polar coordinates, the integral is the length of the paths along the angle of acceptance, divided by the angle of acceptance, doing so we can recover an analytical expression for the average path length. Assuming a uniform distribution of the angles, the integral can be written as

$$\bar{l} = \frac{\int_{\theta_{\min}}^{\theta \max} d(\theta) \mathrm{d}\theta}{\int_{\theta_{\min}}^{\theta \max} 1 \ \mathrm{d}\theta} = \frac{\int_0^1 d(y) \frac{\mathrm{d}\theta}{\mathrm{d}y} \ \mathrm{d}y}{\int_0^1 \frac{\mathrm{d}\theta}{\mathrm{d}y} \ \mathrm{d}y} = \frac{\int_0^L \sqrt{p_x^2 + (y - p_y)^2}^2 \frac{d}{dy}(\tan^{-1}[p_x, y - p_y])dy}{\int_0^L \frac{d}{dy}(\tan^{-1}[p_x, y - p_y])dy}, \tag{36}$$

where $\tan^{-1}$ is the two argument arc tangent. This can be analytically solved for arbitrary $L > 0$ as

$$\bar{l} = \frac{p_x \left( -\log \left( \left( \sqrt{{p_x}^2 + {p_y}^2} - p_y \right) \left( \sqrt{(L - p_y)^2 + {p_x}^2} - L + p_y \right) \right) + \log \left( \sqrt{(L - p_y)^2 + {p_x}^2} + L - p_y \right) + \log \left( \sqrt{{p_x}^2 + {p_y}^2} + p_y \right) \right)}{2 \left( \tan^{-1} \left( \frac{L - p_y}{p_x} \right) + \tan^{-1} \left( \frac{p_y}{p_x} \right) \right)}. \tag{37}$$

## 1.8 Subtended Angle Correction

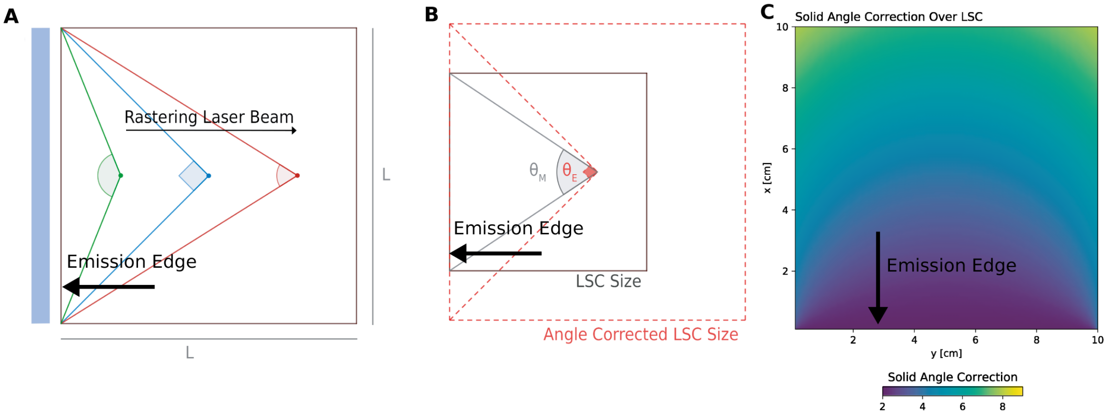


Figure 12: **A** - As the laser beam is rastered across the LSC, the component angle, $\theta_M$ contributing to the measured $\eta_{\text{int}}$ decreases. **B** - Outlines the scenario where the effective angle $\theta_E$ is held at $\frac{\pi}{2}$, the $\eta_{\text{int}}$ as a function of geometric gain can be modelled. **C** - This method can be easily extended to two dimensions as long as the LSC remains square. The distance along the dimensions of the LSC is given by $x$ and $y$, where $y = 0$ corresponds to the emission edge.

As the laser beam is rastered across the LSC, as in **SI Figure 12**, we measure an increasingly smaller solid angle of emission. To correct the PL for a larger LSC, assuming the point we

are exiting is the middle, we multiply by a factor $g$, in the 2D case given by

$$\theta_M = \tan^{-1}(dy, dx_n) + \tan^{-1}((L - dy), dx_n) \tag{38}$$

where $\tan^{-1}$ is defined by the 2 argument arc-tangent, *atan2*, in the euclidean plane. For the case where $dy = \frac{L}{2}$ the expression simplifies to

$$\theta_M = \frac{1}{2}\tan^{-1}\left(\frac{L}{2d}\right), \tag{39}$$

with $\tan^{-1}$ defined in the usual way. The angle of the larger imagined LSC, with the laser beam at its centre, will always have $\theta_E = \frac{\pi}{2}$. Thus, we can write an expression for $g$,

$$\theta_M \; g = \theta_E \rightarrow g = \frac{\pi}{2\theta_M}. \tag{40}$$

The measured optical efficiency, $\eta_{\text{int}}$, multiplied by $g$ will give a lower bound for the efficiency of a LSC with a higher optical gain because the average optical path-length is longer than a uniformly illuminated LSC. The corrected $\eta_{\text{int}}$ is multiplied by 4 to account for the other 3 edges which are covered.

$$\eta_{int,effective} = 4 \; g \; \eta_{\text{int}} \tag{41}$$

## 1.9 Pathlength, Angle Correction and Geometric Gain

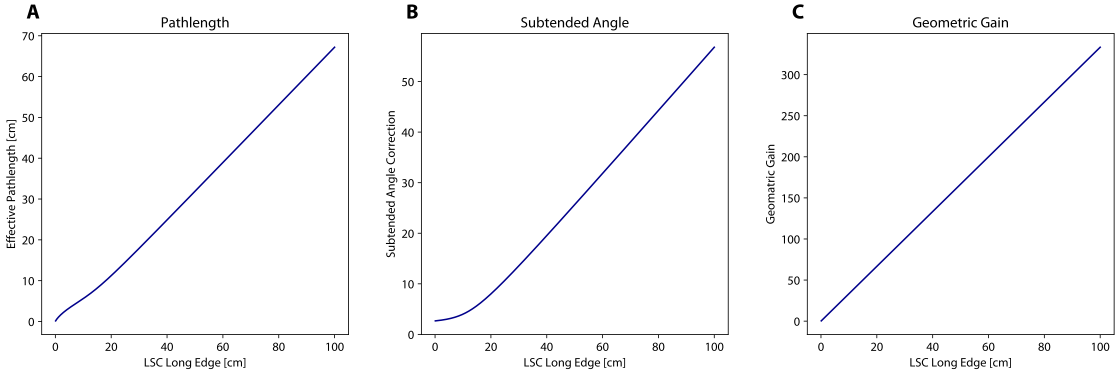


Figure 13: **A** - Pathlength as a function of LSC long edge size, assuming centre illumination and a 10 cm emitting edge. **B** - Subtended angle correction as a function of LSC long edge size assuming a 10 cm by 10 cm with one emitting edge. **C** - Geometric gain as a function of LSC long edge size.

We have determined the effective pathlength as a function of real space position on the measured LSC in **SI Section 1.7**. We also scaled the measured photoluminescence as a function of pathlength by the subtended angle as detailed in **SI Section 1.8**. Doing so generates a dataset of photoluminescence as a pathlength, which can be related to LSC size assuming centre illumination of the imagined LSC. For example, considering the PL at the corner of the measured 10 cm by 10 cm LSC, the effective pathlength is 11.22 cm. The photoluminescence is corrected by a subtended angle correction factor of 8, which corresponds to the centre of a 20 cm by 20 cm LSC. This is extended to arbitrary size in **SI Figure 13**.

Typically LSC sizes are quoted as a factor of geometrical gain, G,,[12] which is the ratio of the top surface aperture area ($A_{\text{active}}$) to the total edge area of LSC covered by PV solar cell ($A_{\text{edge}}$), which may be simplified in the case of a square LSC as

$$G = \frac{A_{\text{active}}}{A_{\text{edge}}} = \frac{A_{\text{long}}^2}{A_{\text{long}} A_{\text{height}}} = \frac{A_{\text{long}}}{A_{\text{height}}}. \tag{42}$$

As the height of the LSC remains fixed, $A_{\text{height}}$ is constant so $G$ is directly proportional to

the long edge of the square LSC.

## 1.10 Single Value Decomposition

SVD facilitates the interpretation of observed spatially resolved photoluminescence by reducing the dimensionality of the problem. Let matrix D describe the measured photoluminescence at the measured pathlengths where D is an $m \times n$ real matrix with $m > n$, where $m$ is the number of pathlengths sampled, $n$ is the number of wavelengths recorded, then D can be written in the form

$$\mathrm{D} = \mathrm{USV}^{\mathrm{T}}. \tag{43}$$

where U has dimensions of $m \times m$, S has $m \times n$, and V has $n \times n$. U and V are unitary, so that $\mathrm{U}^{\mathrm{T}}\mathrm{U} = \mathrm{I}$ and $\mathrm{V}^{\mathrm{T}}\mathrm{V} = \mathrm{I}$ (where the two identity matrices may have different dimensions). S has entries only along the diagonal, known as singular values. The weighted left singular vectors (wLSV) are given by US.

Decomposing the spatial data in this way has a useful interpretation; U is the matrix of left singular vectors giving the spatial dependence of the signal, $\mathrm{V}^T$ is the matrix giving the spectral dependence of the signal as plotted in **Figure 4A** and **B**, respectively. The goal is to determine what subset of the data is required to adequately describe the full dataset. The best practice in choosing SVD components is to target a minimally descriptive model, using the smallest possible set of components to describe the data.[13]

Adapting Beer's law (**Equation 4**) into matrix form, and using the reduced weighted left singular vectors in place of the full data matrix, we can write

$$(\mathrm{US})_n = \mathrm{E}(\vec{\alpha})\mathrm{x}, \tag{44}$$

where $(\mathrm{US})_n$ represents the matrix of chosen weighted left singular vectors. Here E is the

design matrix, which is an exponential function of the absorption coefficients vector, $\vec{\alpha}$. Array x corresponds to the coefficient, $A_i$, for each weighted left singular vector. The problem becomes for what vector of absorption coefficients, $\vec{\alpha}$, is **SI Equation 44** best satisfied, which can be solved efficiently using any good numerical solver, by solving the associated least squares problem,

$$\min_{\vec{\alpha}} \left\| (US)_n - E(\vec{\alpha})x \right\|_2^2 , \tag{45}$$

where the subscript refers to the Euclidean norm. The residue is then the norm of the square of all the differences. If the number of exponentials is not sufficient to describe the measured data, this suggests a number of absorption coefficients higher than the number of components detected by SVD. The number of components need not be equal to the number of spectrally distinct components present.[14]

## 1.11 Determining $\eta_{EQE}$

It is also possible to use the $\eta_{\text{int}}$ values to determine the edge photon flux of the LSC as a function of LSC size. Defining the terrestrial solar spectrum, AM1.5($\lambda$), in units of photons per area per nanometer per unit time, we can calculate the number of photons impinging,$\gamma_{in}$ onto the surface of the LSC,

$$\gamma_{in} = \int_0^\infty t \text{ AM1.5}(\lambda)\ d\lambda, \tag{46}$$

.

where $t$ is the unit of time. The integral of the solar spectrum times the absorption, $\text{Abs}(\lambda) = 1 - 10^{-A(\lambda)}$, per nanometer of the LSC multiplied by the internal efficiency will equal the

number of photons emitted by the edge of the LSC, $\gamma_{\text{emitted}}$, which is a function of LSC size, $G$,

$$\gamma_{\text{emitted}}(G) = \eta_{\text{int}}(G) \underbrace{\int_0^{\infty} t \text{ AM1.5}(\lambda) \text{ Abs}(\lambda) \, d\lambda}_{\text{photons absorbed}} . \tag{47}$$

The wavelength dependence of the emitted photons can be determined as we have an expression for the photoluminescence as a function of arbitrary $G$ in **Equation 4**. Hence

$$\gamma_{\text{emitted}}(G) = x \int_0^{\infty} t \text{ PL}(G, \lambda) \, d\lambda, \tag{48}$$

where $x$ is some scaling factor which relates the normalised PL to the actual number of photons emitted from the edge and can be determined by comparing **SI Equations 47 & 48**. The number of photogenerated charges out, $\text{e}_{\text{out}}$ is dependent on the EQE of the cell, and can be determined from the integral of the scaled PL, now in photons per nm, and the EQE of the cell,

$$\text{e}_{\text{out}} = \int_0^{\infty} x \text{ PL}(G, \lambda) \text{ EQE}(\lambda) \, d\lambda. \tag{49}$$

Then $\eta_{EQE}$ is $\text{e}_{\text{out}}$ divided by $\gamma_{\text{in}}$. Usefully, the same method can be applied readily expanded for arbitrary photon flux. Here we make the explicit assumption that the that the EQE of the cell does not have any power dependence, nor does $\eta_{int}$. Notably, $\eta_{int}$ has been determined using blue laser, with a photon flux much greater than the typical terrestrial solar irradiation.

## 1.12 $V_{OC}$ as a function of concentration

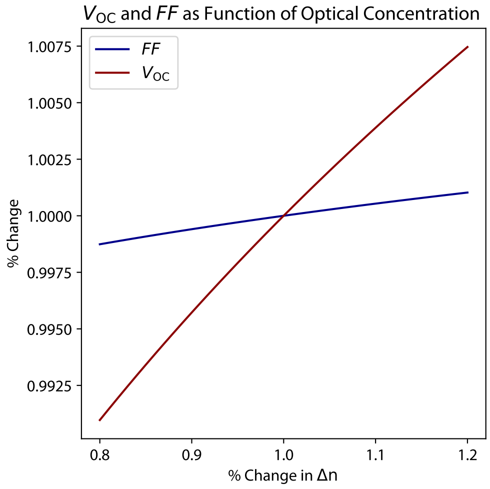


Figure 14: Change in $V_{OC}$ (in red) and $FF$ (in blue) as a function of excess carrier density, representative of change in optical photon flux. Changes are less than 1% over a ±20% range excess carrier density, assuming typical silicon carrier concentrations.

$V_{OC}$ is broadly dependent on the doping ($N_A$), excess ($\Delta n$) and intrinsic ($n_i$) carrier concentrations as

$$V_{OC} = \frac{kT}{q} \ln \left[ \frac{(N_A + \Delta n)\, \Delta n}{n_i^2} \right], \tag{50}$$

where kT/q is the thermal voltage. How the $FF$ changes, is somewhat more complex to determine analytically, but is empirically known to follow in terms of $V_{OC}$ for cases in which the ideality factor, $m$ is known, the series resistance is zero, and the shunt resistance is infinite,[15,16]

$$FF = \frac{\nu_{OC} - \ln(\nu_{OC} + 0.72)}{\nu_{OC} + 1} \tag{51}$$

in which

$$\nu_{\mathrm{OC}} = \frac{V_{\mathrm{OC}}}{(mk_B T/q)}. \tag{52}$$

The ideality factor for a good silicon solar cell is typically close to 1.[16] For carrier densities representing a standard silicon solar cell, this might be expected to vary by less than 1% valid over ± 20 % concentration (see **SI Figure 14**), suggesting the assumption in the main text holds irrespective of photon loss due to poor coupling to the LSC.

# References


(1) Yoon, H. W.; Gibson, C. E.; Eppeldauer, G. P.; Smith, A. W.; Brown, S. W.; Lykke, K. R. Thermodynamic Radiation Thermometry Using Radiometers Calibrated for Radiance Responsivity. *International Journal of Thermophysics* **2011**, *32*, 2217–2229.

(2) Grum, F.; Luckey, G. W. Optical Sphere Paint and a Working Standard of Reflectance. *Applied Optics* **1968**, *7*, 2289.

(3) De Greve, B. *Reflections and Refractions in Ray Tracing*; 2006.

(4) Mooney, J.; Kambhampati, P. Get the Basics Right: Jacobian Conversion of Wavelength and Energy Scales for Quantitative Analysis of Emission Spectra. *Journal of Physical Chemistry Letters* **2013**, *4*, 3316–3318.

(5) Greenham, N. C.; Samuel, I. D.; Hayes, G. R.; Phillips, R. T.; Kessener, Y. A.; Moratti, S. C.; Holmes, A. B.; Friend, R. H. Measurement of absolute photoluminescence quantum efficiencies in conjugated polymers. *Chemical Physics Letters* **1995**, *241*, 89–96.

(6) de Mello, J. C.; Wittmann, H. F.; Friend, R. H. An improved experimental determination of external photoluminescence quantum efficiency. *Advanced Materials* **1997**, *9*, 230–232.

(7) de Clercq, D. M.; Chan, S. V.; Hardy, J.; Price, M. B.; Davis, N. J. Reducing reabsorption in luminescent solar concentrators with a self-assembling polymer matrix. *Journal of Luminescence* **2021**, *236*, 118095.

(8) McDowall, S.; Butler, T.; Bain, E.; Scharnhorst, K.; Patrick, D. Comprehensive analysis of escape-cone losses from luminescent waveguides. *Applied Optics, Vol. 52, Issue 6, pp. 1230-1239* **2013**, *52*, 1230–1239.

(9) Shurcliff, W. A.; Jones, R. C. The Trapping of Fluorescent Light Produced within Objects of High Geometrical Symmetry. *Journal of the Optical Society of America* **1949**, *39*, 912.

(10) Goodman, L. A. On the Exact Variance of Products. *Journal of the American Statistical Association* **1960**, *55*, 708–713.

(11) Ku, H. Notes on the use of propagation of error formulas. *Journal of Research of the National Bureau of Standards, Section C: Engineering and Instrumentation* **1966**, *70C*, 263.

(12) Rafiee, M.; Chandra, S.; Ahmed, H.; McCormack, S. J. An overview of various configurations of Luminescent Solar Concentrators for photovoltaic applications. *Optical Materials* **2019**, *91*, 212–227.

(13) Van Stokkum, I. H.; Larsen, D. S.; Van Grondelle, R. Global and target analysis of time-resolved spectra. *Biochimica et Biophysica Acta - Bioenergetics* **2004**, *1657*, 82–104.

(14) Ruckebusch, C.; Sliwa, M.; Pernot, P.; de Juan, A.; Tauler, R. Comprehensive data analysis of femtosecond transient absorption spectra: A review. *Journal of Photochemistry and Photobiology C: Photochemistry Reviews* **2012**, *13*, 1–27.

(15) Green, M. A. Solar cell fill factors: General graph and empirical expressions. *Solid-State Electronics* **1981**, *24*, 788–789.

(16) Leilaeioun, M.; Holman, Z. C. Accuracy of expressions for the fill factor of a solar cell in terms of open-circuit voltage and ideality factor. *Journal of Applied Physics* **2016**, *120*, 123111.
